# Clinical features, acute complications, and outcome of *Salmonella *meningitis in children under one year of age in Taiwan

**DOI:** 10.1186/1471-2334-11-30

**Published:** 2011-01-27

**Authors:** Hung-Ming Wu, Wan-Yu Huang, Meng-Luen Lee, Albert D Yang, Ko-Ping Chaou, Lin-Yu Hsieh

**Affiliations:** 1 Department of Neurology, Changhua Christian Hospital, Changhua, Taiwan; 2 Pediatrics, Changhua Christian Hospital, Changhua, Taiwan; 3 Neuroradiology, Changhua Christian Hospital, Changhua, Taiwan; 4 Psychiatry, Changhua Christian Hospital, Changhua, Taiwan; 5 Department of Neuropediatrics, Kuang-Tein General Hospital, Taichung, Taiwan; 6 Research center of speech and language disorders, Department of Pediatrics, Changhua Christian Hospital, Changhua, Taiwan; 7 Neuroscience & Psychiatry Research Lab, Changhua Christian Hospital, Changhua, Taiwan; 8 Graduate Institute of Acupuncture Science, China Medical University, Taichung, Taiwan

## Abstract

**Background:**

*Salmonella *meningitis remains a threat to children below two years of age in both developing and developed countries. However, information on such infections has not been well characterized. We analyzed data related to twelve years of experience in order to clarify the comprehensive features of *Salmonella *meningitis in our patients, including admission characteristics, acute complications, and long-term outcome.

**Methods:**

The records of patients with spontaneous *Salmonella *meningitis from 1982 to 1994 were retrospectively reviewed. The long-term outcome was prospectively determined for survivors at school age by the developmental milestones reported by their parents and detailed neurological evaluation along with intelligence, hearing, visual, speech and language assessments.

**Results:**

Of the twenty-four patients, seizures were noted in fifteen (63%) before admission and thirteen (54%) during hospitalization. Acute complications mainly included hydrocephalus (50%), subdural collection (42%), cerebral infarction (33%), ventriculitis (25%), empyema (13%), intracranial abscess (8%), and cranial nerve palsy (8%). Three patients (13%) died during the acute phase of *Salmonella *meningitis. The twenty-one survivors, on whom we followed up at school age, have sequelae consisting of language disorder (52%), motor disability (48%), intelligence quotient < 80 (43%), epilepsy (33%), sensorineural hearing loss (17%), visual deficits (10%), abducens nerve palsy (5%), microcephaly (5%), and hydrocephalus (5%). Overall, good outcome was noted in six (28.6%) of twenty-one survivors, mild sequelae in three (14.2%), moderate in six (28.6%), and severe in six (28.6%).

**Conclusion:**

*Salmonella *meningitis in neonates and infants had a wide spectrum of morbidity and acute complications, leading to a complicated hospital course and subsequently a high prevalence of permanent adverse outcome. Thus, early recognition of acute complications of *Salmonella *meningitis and a follow-up plan for early developmental assessment of survivors are vital.

## Background

*Salmonella *strains are an important pathogen of childhood bacterial meningitis in many developing countries, accounting for 5-13% of acute bacterial meningitis in young children in the 1980s-2000s [1-4]. In developed countries, *Salmonella *meningitis is a rare form (1% or less) of acute bacterial meningitis, but such infections are serious and thus still a threat to children below the age of two [5,6]. In 1907, Ghon reported the first case of *Salmonella *meningitis [7]. The previous reports suggested that *Salmonella *meningitis was associated with a very high prevalence (50-90%) of morbidity, presenting variable complications, and a high mortality rate of up to 50-70% [8-15]. However, comprehensive information regarding *Salmonella *meningitis, including clinical features, complications, and long-term sequelae among survivors has not been well characterized. To clarify these systemic features, particularly long-term outcomes of *Salmonella *meningitis occurring in neonates and infants primarily admitted to Changhua Christian Hospital from 1982 to 1994, the clinical characteristics of the disease present at hospital admission were retrospectively reviewed, and a follow-up assessment of patients at school age was prospectively undertaken.

## Methods

Changhua Christian Hospital is a teaching hospital in central Taiwan that serves as a referral center as well as a primary care facility. From 1982 to 1994, we collected one to three cases with *Salmonella *meningitis annually for a total of twenty six patients. Twenty four of them received first aid and all medical care in our hospital, and the other two were referred to us after partial antibiotic treatment by local medical care. After 1994, only a few cases with *Salmonella *meningitis were collected in our hospital due to public health improvements in Taiwan, and they were referred by local medical care. Since the group of referred cases with *Salmonella *meningitis was small and treated with varied durations of antibiotics before admission, this group was excluded from our study. The medical records of all patients primarily admitted to our hospital from 1982 to 1994 with spontaneous *Salmonella *meningitis were reviewed. They fulfilled the entry criteria for diagnosis by the presence of *Salmonella *species in cerebrospinal fluid (CSF) culture, which further supported infection based on pleocytosis (> 30/mm^3^) with predominant neutrophilia and hypoglycorrhachia. Records were examined for initial clinical presentations, demographic features, laboratory data, acute complications at hospitalization, and antibiotic therapy. In this study, the modified Grady coma scale was used to measure the level of consciousness, classing children on a scale of I to V along a scale of lethargy or irritability (grade I), stupor (grade II), deep stupor (grade III), coma with abnormal posturing (grade IV), and coma without any response (grade V) [16]. Follow-up on surviving cases was performed at pediatric neurology outpatient clinics after discharge. This study was conducted with the approval of the Ethical Research Committee of Changhua Christian Hospital. Written informed consents were obtained from the participants and their parents after full oral and written explanation of the evaluation procedures, and the purpose of the study.

The survivors and/or their parents were asked to complete a questionnaire detailing developmental problems and presence of a seizure disorder. Developmental delay was defined as a child not reaching developmental milestones at the expected times, including motor development, hearing, speech and language. In addition, a pediatric neurologist determined the presence of motor disabilities and sequelae in survivors based on neurological examinations. Motor disability was defined as reduced ability to perform normal human motor functions, such as standing and walking. Hearing function was assessed with conventional behavioral pure-tone audiometry. General intelligence was measured using the Wechsler Intelligence Scale for Children-Revised (WISC-R) [17]. Language ability was assessed with a language assessment test as previously described [18]. Cerebral background activity and epileptiform discharge were detected by electroencephalography (EEG). Cranial computed tomography (CT) scan was also performed to document brain tissue changes following *Salmonella *meningitis.

In addition to considering individual assessment results and events of *Salmonella *meningitis during hospitalization, the overall outcome was classified according to previous reports with a few modifications [19]. These were as follows: (1) death: death was associated with *Salmonella *meningitis; (2) severe adverse outcome: severe cerebral palsy (vegetative state and/or tetraparesis, tetraplegia), profound or severe intellectual impairment (intelligence quotient (IQ) score < 55), profound or severe delayed language development (≥ 3 standard deviations (SD) below the mean on the full score of the preschool language scale), intractable epilepsy, sensorineural hearing loss ≥ 60 dB, and/or bilateral cranial nerve palsy or deficits; (3) moderate adverse outcome: moderate to mild cerebral palsy (hemiparesis or paraparesis), moderate intellectual impairment (IQ score between 55 and 69), moderate delayed language development (< 3 SD and ≥ 2 SD below the mean), sensorineural hearing loss (59 to 40 dB), one side of cranial nerve palsy or deficits, and/or epilepsy; (4) mild adverse outcome: minimal neurological impairment (generalized hyperreflexia and/or clumsiness), mild intellectual impairment (IQ score between 70 and 80), mild delayed language development (< 2 SD and ≥ 1.5 SD below the mean), and/or hydrocephalus without complications; and (5) good outcome: no motor disability, no intellectual impairment (IQ score > 80), and normal language development (< 1.5 SD from the mean).

Data is expressed as mean ± SD or median (range). Clinical features at the time of admission and acute complications of *Salmonella *meningitis were evaluated to find the relevant predictors associated with death, severe adverse outcome, and moderate adverse outcome using the 2-tailed Fisher's exact test (SPSS for Windows, version 15, SPSS Inc, Chicago, IL). *P *value < 0.05 was considered significant.

## Results

### Clinical Characteristics and Acute Complications of *Salmonella *Meningitis

We collected a total of twenty-four patients (six neonates and eighteen infants) with *Salmonella *meningitis, who had not received any treatment for the illness before coming to our hospital. All children with growth of *Salmonella *from CSF also fit the other criteria of the CSF pleocytosis (> 30/mm^3^) with predominant neutrophilia and hypoglycorrhachia for meningitis. The records of all eligible patients presented for *Salmonella *meningitis were fully reviewed and all survivors were completed follow-up. Their basic data at the acute phase of *Salmonella *meningitis is summarized in Table 1. HIV infection and other risk factors for invasive disease were absent in our cases. They were admitted to our hospital within three days after symptom onset. The most common agents were *Salmonella enteritidis *Group D1 (41.7%); others included *Salmonella enteritidis *Group B (12.5%), *Salmonella enteritidis *Group C1 (8.3%), *Salmonella enteritidis *Group C2 (4.2%) and *Salmonella *species (33.3%). Clinical features were similar in both the neonate and infant groups at the time of admission, except that a bulging anterior fontanel appeared to be more common in infants (61%) than in neonates (33%). Seizures were noted in 15 (63%) of the twenty-four patients before admission. Seizures occurred in thirteen (54%) during hospitalization, and ten (77%) of those seizures were not well controlled even though antiepileptic drugs were administrated for > 48 hours. Six (25%) had no seizures noted in the course of *Salmonella *meningitis. The laboratory findings for the twenty-four patients are listed in Table 2. Cerebrospinal fluid (CSF)/blood glucose ratio < 0.5 was 83% of those cases and CSF protein levels > 200 mg/dl in 54% of the patients.

**Table 1:** Admission features and progress of the twenty-four patients with *Salmonella *meningitis

Characteristics	Cases (N = 24) n (%)
Male: Female	15: 9
Age at onset, days*	79 ± 44.5 (range, 5 to 266)
Onset of symptoms before admission, days*	2.1 ± 0.74 (range,1 to 3)
Clinical features present between onset of symptoms and day of diagnosis	
Fever	24 (100)
Lethargy	6 (25)
Irritability	10 (42)
Poor feeding	20 (83)
Diarrhea	8 (33)
Vomiting	9 (38)
Bulging anterior fontanel	13 (54)
Nuchal rigidity	9 (38)
Seizure	15 (63)
*Salmonella *isolates	
CSF	24 (100)
Blood	13 (59)
Stool	3 (17)
Urine	1 (6)
Antibiotics	
CM	1(4)
AC, CM	2 (8)
AC, third-generation cephalosporins	6 (25)
CM, third-generation cephalosporins	4 (17)
AC, CM, third-generation cephalosporins	2 (8)
AG, third-generation cephalosporins	2 (8)
Third-generation cephalosporins	7 (29)
Duration of hospitalization, days*	32.1 ± 12.2 (range, 14 to 74)
Condition at discharge	
Survival	21 (88)
No disability	8 (33)
Disability	13 (54)
Ongoing seizure	7 (29)
Focal motor weakness	10 (42)
Cranial nerve palsy	2 (8)
Death	3 (13)
Relapse	1 (4)

*Values are presented as mean ± SD with range in parentheses; n (%), case number with percentage of the patients with positive findings in parentheses; ^#^Denotes CSF cultures done in 24 cases, blood cultures in 22, urine cultures in 16, and stool cultures in 18; third-generation cephalosporins: cefotaxime, moxalaxtam, ceftriaxone, and ceftazidime; AC, Ampicllin; AG, aminoglycoside; CM, Chloramphenicol; disability, impairment of physical ability and seizure.

**Table 2:** Analysis of peripheral leukocytes and CSF at the time of diagnosis

Findings	Cases (N = 24) n (%)
Peripheral blood data	
Total WBC/μl*	12210 (1570-28900)
Segmented WBC/μl*	4760 (527-17138)
Banded WBC/μl*	389 (0-4624)
Platelet (×10^4^/μl)*	32.7 (7.4-87.5)
CSF data	
Total WBC/μl*	1852 (68-30600)
PMN/μl*	1490 (65-24480)
Glucose (mg/L)*	15 (0-69)
Protein (mg/dl)*	330 (40-740)
CSF/blood glucose ratio < 0.5	20 (83)
CSF protein > 200 mg/dl	13 (54)
Gram stain positive	24 (100)

* Values are presented as median (complete range); WBC, white blood cell; PMNs, polymorphonuclear leukocytes.

Initial antibiotics that covered *Salmonella *species were used for the twenty-four patients, based on the results of gram stains and cultures of blood and CSF. They were treated with fourteen different antimicrobial regimens, and most received chloramphenicol or ampicillin in combination with one of the third generation cephalosporins (cefotaxime, moxalaxtam, ceftriaxone, and ceftazidime) (Table 1). In vitro sensitivities to the third generation cephalosporins, chloramphenicol, and ampicillin were 100%, 54.5%, and 45.8%, respectively. Antimicrobial therapy durations were 32.1 ± 12.2 days (range, 14 to 74 days). One case had acute complications, including refractory seizures, ventriculitis, and severe hydrocephalus, and received a ventriculoperitoneal shunt. This patient had a relapse of *Salmonella *meningitis with the same serotype of *Salmonella *33 days after treatment with susceptible antibiotics for 56 days.

During hospitalization, brain sonography (N = 22) and/or CT scan (N = 18) and/or ventricular tap (N = 12) were performed to detect acute intracranial complications and to diagnose ventriculitis. Those complications included hydrocephalus (50%), subdural collection (42%), cerebral infarction (33%), ventriculitis (25%), empyema (13%), intracranial abscess (8%), and cranial nerve palsy (8%) (Table 3). Of twelve cases with hydrocephalus, six had ventriculitis, and three with severe non-communicating type received a ventriculoperitoneal shunt during hospitalization. Eight patients had cerebral infarction and most of them had one or more associated and concomitant complications, mainly hydrocephalus (50%), seizures (75%), subdural effusion (40%), empyema and brain abscess (25%). Four children were found to have focal intracranial infection: two had subdural empyema, one had subdual empyema plus brain abscess, and one had multiple abscesses. All four patients also had other associated and concomitant complications such as prolonged seizures (100%), cerebral infarction (50%), ventriculitis (50%), and abducens nerve palsy (25%). They all received surgical management to drain brain abscess and empyema, and had severe neurological disability at discharge.

**Table 3:** The association of clinic features, CSF findings, and acute complications with the outcome in twenty-four children with *Salmonella *meningitis

Variable	Death, moderate and severe sequelae group (N = 15) n (%)	Mild sequelae and good outcome group (N = 9) n (%)
Level of consciousness		
Lethargy or irritability at admission*	13 (87)	3 (33)
Coma in hospital^#^*	10 (67)	0 (0)
Seizures		
Seizure before admission	10 (67)	5 (56)
Seizure in hospital*	12 (80)	1 (11)
Laboratory tests		
CSF: blood glucose < 0.5*	15 (100)	5 (56)
CSF protein > 200 mg/dl*	11 (73)	2 (22)
Intracranial complications		
Subdural collection	6 (40)	4 (44)
Intracranial focal infection*	5 (33)	0 (0)
Empyema	3 (20)	0 (0)
Brain abscess	2 (13)	0 (0)
Cerebral infarction*	8 (53)	0 (0)
Ventriculitis*	6 (40)	0 (0)
Hydrocephalus	9 (60)	3 (33)

*Denotes a significant difference between death, moderate and severe sequelae group and mild adverse and good outcome group in the univariate analysis using Fischer's exact test (*p *< 0.05); ^#^Denotes Grady coma scale IV and V.

### Outcome of Patients with *Salmonella *Meningitis for School-Age Survivors

Overall, good outcome was observed in six (28.6%) of twenty-one survivors, mild adverse outcome in three (14.2%), moderate in six (28.6%), and severe in six (28.6%). Table 3 summarizes the relevant unfavorable factors associated with outcome. There was a higher prevalence of acute complications and decreased level of consciousness in children who had poor outcome than in those with mild adverse and good outcomes.

Three (13%) of the twenty-four patients with *Salmonella *meningitis died during hospitalization. The remaining twenty-one survivors were prospectively followed up when they were at school age (median 9.4, range 7.8 to 12.2 years) and then evaluated (Table 4). Developmental delay occurred in fourteen (67%) cases. Nearly half (48%) of the survivors had motor disabilities. Among five patients who had severe disabilities, two had spastic tetraplegia with/without urine and stool incontinence, and three had spastic tetraplegia in a vegetative state. Among four with moderate impairment, three had spastic hemiparesis and one had spastic paraparesis. One patient with mild impairment showed slight clumsiness and generalized hyperreflexia. In addition, ataxia was noticed in two patients with hemiparesis, and involuntary movement was noticed in three patients with tetraplegia.

**Table 4:** Long-term outcome associated with acute main complications for *Salmonella *meningitis in twenty-one survivors

Findings	Survivors (N = 21) n/total (%)*	Number of acute main complications in each particular outcome						
		Seizure^#^	Sudural effusion	Hydro- cephalus	Ventr-iculitis	Em-pyema	Brain abcess	Cerebral infarction
Development								
Delayed	14/21 (67)	11	5	8	5	3	2	7
Normal	7/21 (23)	0	4	3	0	0	0	0
Motor disability								
Severe	5/21 (24)	5	2	5	5	1	2	3
Moderate	4/21 (19)	3	3	1	0	1	0	4
Mild	1/21 (5)	0	0	0	0	0	0	0
Normal	11/21 (52)	3	4	4	0	1	0	0
Hearing Problems	3/18 (17)	2	1	3	1	1	1	0
Epilepsy	7/21 (33)	6	2	4	4	3	2	5
Microcephaly	1/21 (5)	1	0	1	1	0	1	1
Hydrocephalus	1/21 (5)	0	0	0	0	0	0	0
Visual deficit	2/21 (10)	1	2	1	0	0	0	1
CN6 palsy	1/21 (5)	0	1	1	0	0	0	0
Intelligence								
IQ < 55	6/21 (29)	6	2	5	5	2	2	4
IQ 55-69	1/21 (5)	1	0	0	0	1	0	0
IQ 70-80	2/21 (10)	1	1	1	0	0	0	1
IQ > 80	12/21 (57)	3	6	5	0	0	0	2
Speech/language								
Abnormal	11/21 (52)	9	4	7	5	3	2	6
Normal	10/21 (48)	2	5	4	0	0	0	1

*Values are expressed as number of patients with each particular outcome/number of patients examined, and numbers in parentheses are percentages; ^#^Seizure in hospital; CN6, Abducens nerve palsy; MR, mental retardation; IQ, intelligence quotient.

Seven (33%) cases of the twenty-one survivors developed epilepsy. Five had refractory epilepsy that developed at the time of their acute illness, and the other two developed epilepsy post *Salmonella *meningitis at two and four years. All seven cases had focal or multifocal epileptiform discharges on EEG recordings. In addition, one survivor who did not display clinical symptoms of seizure had abnormal focal epileptiform discharges.

In sixteen (76%) of twenty-one children, brain CT scans revealed abnormal findings, suggesting old infarction and lesions associated with acute *Salmonella *meningitis. Moderate hydrocephalus was detected in one child, who had generalized tendon hyperreflex and good intelligence. Another patient who had received a ventriculoperitoneal shunt at age six months due to severe hydrocephalus had a normal sized ventricle.

One (5%) of twenty-one children had had left abducens nerve palsy since *Salmonella *meningitis and two (10%) had visual deficits. Sensorineural hearing impairment was noticed in three (17%) of the eighteen survivors tested by pure-tone audiometry. Two had severe unilateral sensorineural loss (> 60 dB) while one had mild unilateral sensorineural loss (40 dB). The other three survivors could not be tested due to their severe disabilities.

The language assessment test for school-age children was designed to identify language comprehension and verbal expression ability [18]. Of the twenty-one survivors who completed the test, eleven (52%) were found to have speech and language developmental delay, of whom five had severe communication problems or absence of speech. Thus, these five could not perform the neuropsychological tasks on the WISC-R, and the estimated score for each was assigned a poor performance under -3 SD for age because of their severe intellectual disabilities. The other sixteen children completed the WISC-R. The median full-scale IQ for these 16 cases was 93.5 (range, 48 to 107). Two children had borderline learning difficulty and another two had a moderate learning difficulty or worse.

Table 3 summarizes the relevant predictors associated with death and severe to moderate adverse outcomes found by univariate analyses. The major unfavorable factors presented during *Salmonella *meningitis were conscious change, seizure during hospitalization, CSF/blood glucose ratio < 0.5, CSF protein > 200 mg/dl, focal intracranial infection, ventriculitis, and cerebral infarction. The features including seizure onset before admission, subdural collection, and hydrocephalus were not significantly associated with a poor long-term outcome.

## Discussion

In this study, three out of twenty-four patients with spontaneous *Salmonella *meningitis died, representing a mortality rate of 13%; eighteen (75%) cases developed at least one complication at the acute phase of *Salmonella *meningitis, leading to a complicated clinical course. At school-age follow-up, fifteen (71%) of the twenty-one survivors had subsequent motor disabilities, epilepsy, language delay, and intelligence impairment (Table 4), and most (58%) suffered from moderate to severe sequelae. We further revealed that death and major adverse outcomes were highly associated with seven unfavorable factors during *Salmonella *meningitis: conscious change, seizure during hospitalization, CSF/blood glucose ratio < 0.5, CSF protein > 200 mg/dl, focal intracranial infection, ventriculitis, and cerebral infarction (Table 3)

The incidence (71%) of mild to severe adverse long-term outcomes in our twenty-one survivors was high, compared with those in the previous studies [8-13,15]. One explanation is that our cases demonstrated more complicated involvement of the brain, such as cerebral infarction. On the other hand, our children were followed up at school age. Almost all previous studies involved follow-up of children aged less than five years [8-13,15]. Since survivors remain at high risk for neurologic sequelae and lifelong impairment following meningitis [20,21], there is difficulty in assessing those cases, particularly during the neonate period or during the first two years of life. Our study may thus provide a more accurate assessment for survivors, and give an incidence of adverse long-term outcome resulting from *Salmonella *meningitis.

Previous studies have demonstrated many prognostic factors for an unfavorable outcome of bacterial meningitis in children, such as *Streptococcus pneumonia*e meningitis [2,22]. Since *Salmonella *meningitis is a relatively rare form of bacterial meningitis in neonates and infants, until now, there have been few studies that focus on the predictive factors for long-term outcomes [10,12]. Although our case number was not so large, this study does allow us to assess trends in the distribution of an adverse outcome in relation to factors discernible at the acute phase of *Salmonella *meningitis (Table 3). The group with major adverse outcome and death was highly associated with seven unfavorable factors in comparison to the group with mild to no adverse outcome in univariate analysis (*p *< 0.05, Fisher's exact test), e.g. conscious alteration, late seizures, high CSF protein, low CSF/blood glucose ratio, cerebrovascular involvement, and intracranial infectious complications (Table 3). These factors are consistent with most predictive factors found in other bacterial meningitis [2,22].

Treatment of *Salmonella *meningitis has not been well defined. Antimicrobial agents, including chloramphenicol, ampicillin, and cotrimoxazole, had a low cure rate (~ 40%) and a highly associated mortality (~ 45%) [23]. Third-generation cephalosporins and fluoroquinolones had a high cure rate (> 80%) and lowered the associated mortality (< 10%) [10,24-26]. Thus, those two antibiotics would be regarded as the optimal antimicrobial regimens in the treatment of *Salmonella *meningitis [26]. All our patients were treated initially with antibiotics to which their *Salmonella *species was susceptible, and most received third-generation cephalosporins, combined with chloramphenicol or ampicillin. Similar to the previous report [10], the mortality rate in our cases declined to about 10%, but the morbidities remained high, up to 75%. The results suggest that susceptible antibiotics for treatment of *Salmonella *meningitis in children were unsatisfactory. Adjunctive dexamethasone, therefore, may be recommended to attenuate the effects of the acute innate inflammatory response to bacterial invasion within the CNS [27,28], although the benefits of steroids in meningitis seemed controversial in developing countries [29]. In addition, the patient should be monitored in the intensive care unit for any morbidity, such as seizure, in order to detect its potential complication.

In the present study, there are some limitations that merit discussion. The admission features of our patients were retrospectively collected, and the sample size was not large enough for a good statistical analysis. The assessment methods were not fully validated. For instance, a standard clinical examination was performed to identify children with a gross motor impairment, which might not fully assess motor function in those with minimal deficits. Nevertheless, about a half of our postmeningitic children followed up at school age had neuromuscular disability, which was high in prevalence among bacterial meningitis [30-32]. Second, the study was limited by the lack of a sibling control group for comparison, since the possibility that some of the cognitive impairment detected might not be attributive to *Salmonella *meningitis must be considered. However, each of the tests used for neuropsychological and language assessment had been validated for normal populations, providing assurance that the intellectual deficits noted were the result of *Salmonella *meningitis. Our children with moderate to profound MR had more risk factors (5.2 ± 0.5) than those with normal average IQ and mild MR (1.6 ± 1.1) (Table 3). Thus, the results suggest that their motor disabilities and cognitive impairment might be highly related to the severity of their medical condition during the acute phase.

## Conclusions

In summary, the present study provides more insight into a clinical spectrum and long-term outcome in the patients with *Salmonella *meningitis. Our study further revealed that *Salmonella *meningitis resulted in a high prevalence of long-term adverse outcome, being linked with its insult of acute complications, and a broad range of the potential neurological sequelae after meningitis remained with age. Early recognition of acute complications of *Salmonella *meningitis and a follow-up plan for early developmental assessment of survivors are vital.

## Abbreviations

CSF: cerebrospinal fluid; EEG: electroencephalography; IQ: intelligence quotient; WISC-R: Wechsler Intelligence Scale for Children-Revised.

## Competing interests

The authors declare that they have no competing interests.

## Authors' contributions

HMW was responsible for the design, the concept and the writing of this manuscript. WYH and MLL contributed to data collection and conducted the primary analysis. ADY conducted image analysis. KPC contributed to speech and language assessment and its analysis. LYH contributed to neuropsychological assessment and its analysis. All authors read and approved the manuscript.

## Pre-publication history

The pre-publication history for this paper can be accessed here:

http://www.biomedcentral.com/1471-2334/11/30/prepub
